# A dynamic multi-objective optimization method based on classification strategies

**DOI:** 10.1038/s41598-023-41855-2

**Published:** 2023-09-14

**Authors:** Fei Wu, Wanliang Wang, Jiacheng Chen, Zheng Wang

**Affiliations:** 1 College of Computer Science and Technology, Zhejiang University of Technology, HangZhou, ZheJiang 310023 China; 2 School of Computer and computational Sciences, Zhejiang University City College, Hangzhou, Zhejiang 310015 China

**Keywords:** Evolution, Evolutionary theory

## Abstract

The dynamic multi-objective optimization problem is a common problem in real life, which is characterized by conflicting objectives, the Pareto frontier (PF) and Pareto solution set (PS) will follow the changing environment. There are various dynamic multi-objective algorithms have been suggested to solve such problems, but most of the methods suffer from the inability to balance the diversity of populations with convergence. Prediction based method is a common approach to solve dynamic multi-objective optimization problems, but such methods only search for probabilistic models of optimal values of decision variables and do not consider whether the decision variables are related to diversity and convergence. Consequently, we present a prediction method based on the classification of decision variables for dynamic multi-objective optimization (DVC), where the decision variables are first pre-classified in the static phase, and then new variables are adjusted and predicted to adapt to the environmental changes. Compared with other advanced prediction strategies, dynamic multi-objective prediction methods based on classification of decision variables are more capable of balancing population diversity and convergence. The experimental results show that the proposed algorithm DVC can effectively handle DMOPs.

## Introduction

Dynamic multi-objective optimization problems (DMOPs)^1^ are common practical problems that typically have multiple conflicting objectives. At present, most multi-objective optimization solutions^2–4^ are used to solve static optimization problems^5–8^. Nevertheless, a reality multi-objective optimization problem is a complex optimization problem with a dynamic environment, changing parameters, conflicting objectives. Due to the constantly changing environment, the Pareto optimal front (POF) and the Pareto optimal set (POS) are not fixed. Static optimization methods have been widely used in the field of evolutionary computation, but they often suffer from the lack of fast response mechanisms. In particular, the populations in Dynamic Multi-Objective Optimization Problems (DMOPs) tend to lose diversity over time, which results in a gradual convergence of the populations at later stages of evolution. This convergence makes it increasingly difficult for the populations to adapt to new environments^9^. To address this issue, it is important to develop improved Multi-Objective Evolutionary Algorithms (MOEAs)^10,11^ that have fast response mechanisms, which can help to maintain the diversity of populations and ensure their adaptability to changing environments. In recent years, many dynamic multi-objective algorithms have been proposed, and these methods can be roughly divided into: diversity introduction methods, diversity maintenance methods, prediction-based dynamic multi-objective methods, and classification based on decision variables.

The ultimate goal of the diversity introduction method^12^ and the diversity maintenance method^13^ is to increase the diversity of the population. Some mutant individuals are introduced into the population. With the help of mutant individuals, the population can better follow the environment changes and converge, instead of falling into convergence prematurely and unable to adapt to the environment changes. However, diversity-based methods have very significant disadvantages. Diversity-based methods rely too much on static algorithms, and can only find new optimal solution sets through static algorithms, so the convergence speed will continue to decline. Therefore, how to stop relying on static methods to find diversity points and how to add diversity points to make the population reach the ideal state?

The prediction-based dynamic multi-objective approach^14^ uses predictive models to predict quality populations after changing environments. Although, predictive models play a role in the convergence of populations. Nevertheless, most predictive models require training cycles and the performance of predictive models on certain problems is sometimes generally poor. Consequently, the predictive models required for prediction-based methods need to have two conditions: 1. They need to have a short training cycle and be able to respond quickly; 2. For predictive models to be robust they need to be able to cope with different types of test problems.

The classification of decision variables^15^ can be divided into three broad ways: (1) dominance relationship-based classification of decision variables^16^. At its core, the idea is to perturb the detected decision variables and observe whether all perturbations are mutually dominant. If all perturbations are not mutually dominant, then the decision variables are defined as diversity correlated. All cases other than this are defined as convergence-related. Adopting this category usually causes populations to be overly enmeshed in dominance relationships at the expense of population diversity. (2) Angle-based classification of decision variables^17^. A fitting line is formed using the perturbation points, and the angle between the fitting line and the hyperplane normal is judged by the angle between the fitting line and the hyperplane normal. An acute angle is close to a right angle, then the decision variables are defined as diversity related, otherwise they are defined as convergence related. (3) Monotonic-based classification of decision variables^18^. The method defines positive and negative correlations between decision variables and objectives. And on this basis, the Spearman rank correlation coefficient (SRCC)^19,20^ is used to evaluate the correlation between variables and objectives to avoid the defect of dominance relationship failure. These three approaches to categorizing decision variables all suffer from the same problem: how to design different evolutionary strategies to address different decision variables?

In summary, the dynamic optimization algorithm design needs to face the following problems:

1. How to introduce diversity correctly and keep the population convergence and diversity balanced?
2. In order to respond quickly to environmental changes, what is the best way to design a prediction strategy that is both robust and efficient?
3. If the decision variables are classified, what kind of method should be used and what is the response strategy after classification?

Based on the above problems, this paper proposes a dynamic multi-objective optimization method based on the classification of decision variables. The classified decision variables can better predict the populations after environmental changes. Different prediction strategies are effective in balancing the diversity and convergence of populations. The main contributions of this paper are as follows:

- A static classification strategy is proposed to classify decision variables into three categories, which enables the algorithm to better identify the uses of decision variables. The different decision variables are explored more effectively to balance the diversity and convergence of the new middle group.
- One strategy is proposed to correct the classification of decision variables in a dynamic environment. The population is prompted to respond to environmental changes and adjust the evolutionary direction of decision changes to ensure that the population evolves in the right direction.
- Different prediction strategies are provided for different decision variables, and adaptive prediction strategies are selected according to different decision variable categories, thus enabling adaptation to dynamic environments. (3) Different prediction strategies are provided for different decision variables, and adaptive prediction strategies are selected according to different decision variable categories, thus enabling adaptation to dynamic environments.

## Related work

DMOPs are defined as minimization problems, and their mathematical formulation can be defined as follows:

1 $$\begin{aligned} \begin{aligned} \min \ F\left( x,t \right) =\left( f_1\left( x,t \right) ,\ldots ,f_m\left( x,t \right) \right) ^T \\ s.t.{\left\{ \begin{array}{ll} h_i\left( x,t \right) =0,\ i=1,2,\ldots ,n_h\\ g_i\left( x,t \right) \geqslant 0,\ i=1,2,\ldots ,n_g\\ \end{array}\right. } \end{aligned} \end{aligned}$$

where $x=\left( x_1,x_2,\ldots ,x_n \right)$, represent the *n*-dimensional decision variables. *m* is the number of objectives, $h_i$ and $g_i$ represent the equation and inequality constraint, correspondingly. $n_h$ and $n_g$ represent the number of constraints, respectively.The variable *t* included in the objective function of DMOPs represents the time variable, which is calculated as follows:

2 $$\begin{aligned} t=\frac{1}{n_t}\Bigg \lfloor \frac{\tau }{\tau _t} \Bigg \rfloor \end{aligned}$$

The value of *t* is related to the number of iterations of EA, so $\tau$ in the formula represents the number of iterations, which directly affects the value of *t*.

### Definition 1

(*Dynamic Pareto Dominance*) At time *t*, assume that any two individuals $x_1$ and $x_2$ in the population satisfy the condition:

3 $$\begin{aligned} \begin{aligned} \forall i\in \left\{ 1,\ldots , m \right\} :\ f_i\left( x_1,t \right) \leqslant f_i\left( x_2,t \right) \\ \wedge \exists j\in \left\{ 1,\ldots m \right\} :\ f_j\left( x_1,t \right) <f_j\left( x_2,t \right) \end{aligned} \end{aligned}$$

consider that individual $x_1$ dominates individual $x_2$, written mathematically as: $x_1\prec x_2$.

### Definition 2

(*Dynamic Pareto-Optimal Set*) At time *t*, assume that there is no decision vector $x^{\prime }\in R^n$ dominating *x*, then *x* is a pareto optimal solution, the set of all non-dominated solutions is called the Pareto-Optimal set (POS), that is:

4 $$\begin{aligned} POS_t=\left\{ x\in R^n|\lnot \exists x^{\prime }\in R^n,\ x^{\prime }\prec x \right\} \end{aligned}$$

### Definition 3

(*Dynamic Pareto-Optimal front*) At time *t*, the mapping of the $POS_t$ in the objective space is called $POF_t$ expressed as:

5 $$\begin{aligned} POF_t=\left\{ F\left( x,t \right) |\ x\in POS_t \right\} \end{aligned}$$

According to the different combinations of dynamic changes in the *POF* and *POS*, identified four different types of DMOPs in Table 1.

**Table 1:** DMOPs in different forms.

TYPE	POS	POF
I	Change with time	Remain the same
II	Remain the same	Change with time
III	Change with time	Change with time
IV	Remain the same	Remain the same

In practical scenarios, when the environment changes, the four types of changes mentioned above may occur simultaneously, and we mainly study the first three types of changes. Dynamic multi-objective evolutionary algorithms generally have the following steps:

- **Step 1:** Initialize the population and set the relevant parameters.
- **Step 2:** Detect environmental changes, if the environment changes, go to **Step 4**. If not, go to **Step 3**.
- **Step 3:** Optimize the population using an evolutionary algorithm.
- **Step 4:** Adopt certain response strategies, such as diversity maintenance, diversity introduction, prediction mechanism and memory mechanism, to cope with the changes in the environment.
- **Step 5:** Determine the termination condition, if it is not met, go to **Step 2**, and if it is met, exit.

### Diversity introduction method

In recent years, various DMOAs have been proposed to address DMOPs, and the core idea of these DMOAs is to balance diversity and convergence in response to environmental changes. The first consideration after environmental changes is to maintain the diversity of the population and to introduce random or mutant individuals in relation to the detection of environmental changes, so the introduction of diversity becomes one of the solutions. The introduction of diverse individuals cannot be introduced randomly, as the dynamic problem Pareto solution set and Pareto frontier change with the environment. In other words, increasing population diversity needs to be added through theoretical methods, while blindly adding random points will only generate populations in a bad direction. Consequently, Deb et al.^21^ introduced diverse individuals by tracking the Pareto frontier according to this feature, and the new population formed can be better adapted to the population. An extended algorithm for dynamic vector evaluation particle swarm optimization (VEPSO) was proposed by Harrison et al.^22^ to address the shortcoming that the change detection mechanism relies on the observation of changes in the target space. Based on NSGA-II^23,24^, the DNSGA-II^25^ algorithm was proposed, which was divided into two adaptive algorithms, DNSGA-II-A^26^ as well as DNSGA-II-B, but was not conducive to solving complex environmental change problems.

### Diversity maintenance methods

The diversity maintenance approach, which can also be called a memory-based strategy, focuses on using historical information in the stored environment and reusing them after the environment changes. In contrast to diversity introduction methods, diversity maintenance mechanisms usually directly store historical PS as initialized populations. Nevertheless, this method is limited to solving continuum optimization problems, and it is also difficult to cope with high degree of environmental changes. Li et al.^27^ proposed a novel dynamic multi-objective optimization algorithm (DMOA-DM) based on region local search and memory, using NSGA2-DM to store useful information (memory) to guide the optimization of populations. Liang et al.^28^ proposed a novel dynamic multi-objective evolutionary algorithm which incorporates a hybrid algorithm of memory and prediction strategy (HMPS)^29,30^ and decomposition-based multi-objective evolutionary algorithm (MOEA/D^31^). Differential prediction based on two consecutive population centroids is utilized if the detected change is not similar to any historical change; otherwise, memory-based techniques are applied to predict the new position of the population.

### Prediction-based methods

The prediction-based dynamic multi-objective optimization algorithm also exploits some historical information, but in contrast to the diversity maintenance approach, the prediction-based approach is more reliant on the prediction strategy, and the optimal solution after the change of environment is dependent on the strength of the prediction model. Unfortunately, this method only takes into account changes in the center of the flow pattern and is only relevant to the historical information of the last moment, so it has significant limitations for population updating. Zhou et al.^32^ proposed a population prediction strategy (PPS) that can initialize the entire population by combining the prediction center and the estimated stream shape. Zou et al.^33^ combined special points and centroids to create a prediction mechanism. This prediction mechanism uses the adjacent time interval as the prediction step and directly predicts the set of non-dominated solutions. Jiang et al.^34^ developed an inflection point-based migration learning method called KT-DMOEA. in the proposed method, a trend prediction model (TPM) was developed to generate predicted inflection points, and then an unbalanced migration learning method along with the TPM predicted inflection points were used to generate high-quality initial populations.

### Decision variable classification methods

The methods of diversity introduction as well as the prediction methods do not consider the properties of the decision variables during the iteration, and these methods can be regarded as a probabilistic model for searching for optimal values of the decision variables. In other words, these methods assume that all decision variables have the same probability of functioning in diversity as well as convergence on the population. Nonetheless, in most DMOPs, the probabilities are different, and different nature of decision variables should be corresponded to different search models to get better solutions. In static MOPs, the categories of decision variables can be determined by perturbing the decision variables to produce a large number of individuals and then using fitness assessment^35–38^. While the decision variable classification in static MOEAs is performed only once and the categories of decision variables will not revert after classification, for dynamic MOPs these strategies lose their effectiveness. Liang et al.^39^ proposed a dynamic multi-objective evolutionary algorithm based on the classification of decision variables. The Spearman rank correlation coefficient (SRCC)^18^ was used to determine the categories of the decision variables in static classification. A nonparametric *t*-test was utilized to further correct for variable categories after environmental changes when dynamic environments were in place. Liu et al.^16^ introduced a new DVC classification method for decision variables based on the monotonicity of the optimization objective, which does not make use of dominance relations but uses reference vectors to guide the analysis of decision variables.

## Proposed algorithm

**Figure 1:**
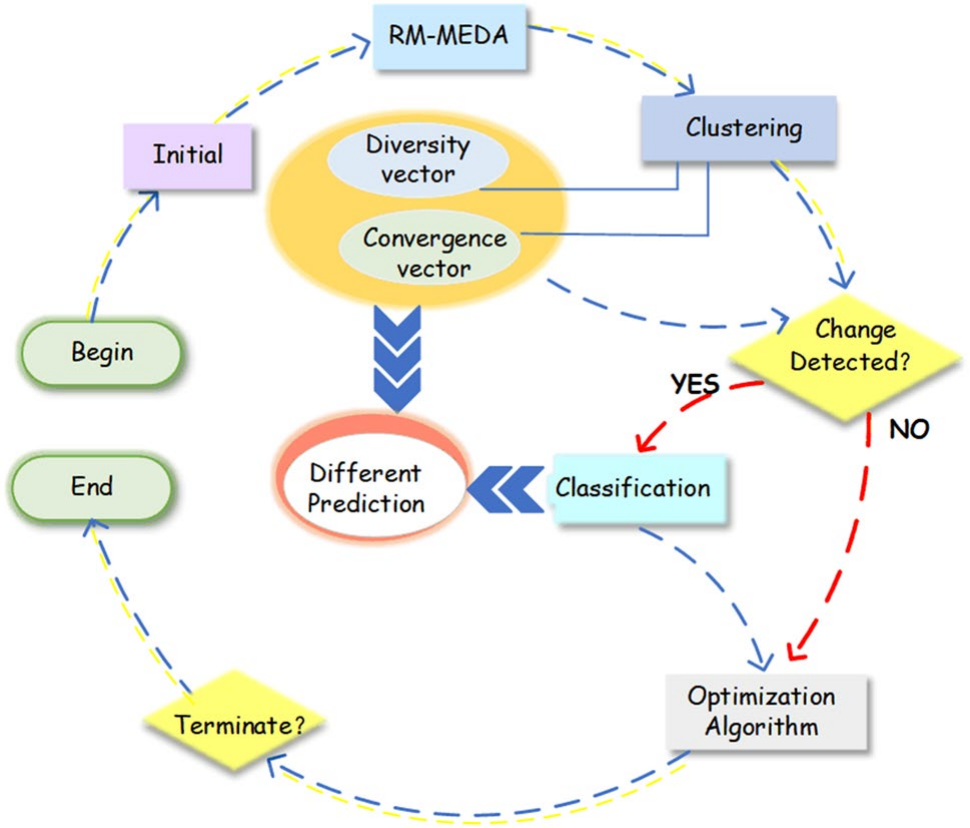
General flowchart of the algorithm.

To address the above problems, we propose a dynamic multi-objective optimization algorithm based on classification prediction. The algorithm consists of three important parts: The first part is the classification strategy of static decision variables, which divide decision variables into two categories: diversity-related decision variables and convergence-related decision variables. The diversity-related decision variables will be an important reference after environmental changes to prevent the population from falling into a local optimum and converging prematurely. The second part is the dynamic classification adjustment strategy after the environment changes, which further determines the predictability of the decision variables. The third part uses different prediction strategies based on the results of the dynamic classification respectively. The components of the algorithm are shown in Fig. 1.

### RM-MEDA

Under relaxed conditions, the Karush-Kuhn-Tucker criterion^40^ demonstrates that the POS of a continuous multi-objective optimization problem is a segmentally continuous $\left( m-1 \right)$-dimensional manifold. The algorithm works as algorithm 1:
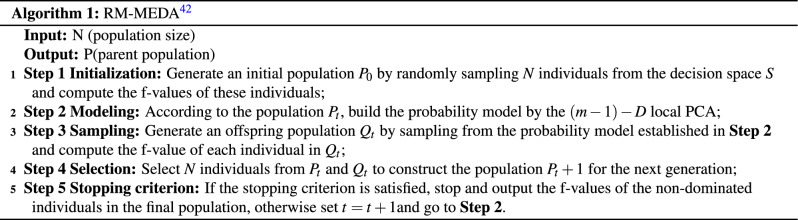


### Classification strategy in static environment

Classification with static decision variables in DMOEA helps populations to be able to search for quality individuals quickly when the environment changes. DMOEA ought to have not only efficient convergence performance but also good distributivity on PF. Nevertheless, the dynamic environment limits the diversity of populations, and early convergence and falling into local optima are common problems of DMOPs. The problem of classification of decision variables is mentioned in the literature^42^, where decision variables are perturbed and divided into three different categories according to different dominance: (a) The relationship between the representative solution and the disturbance solution is nondominated; (b) The relationship between the representative solution and the perturbation solution is dominated; *c)* Both dominated and nondominated relations exist between the representative solution and the perturbation solution.

**Figure 2:**
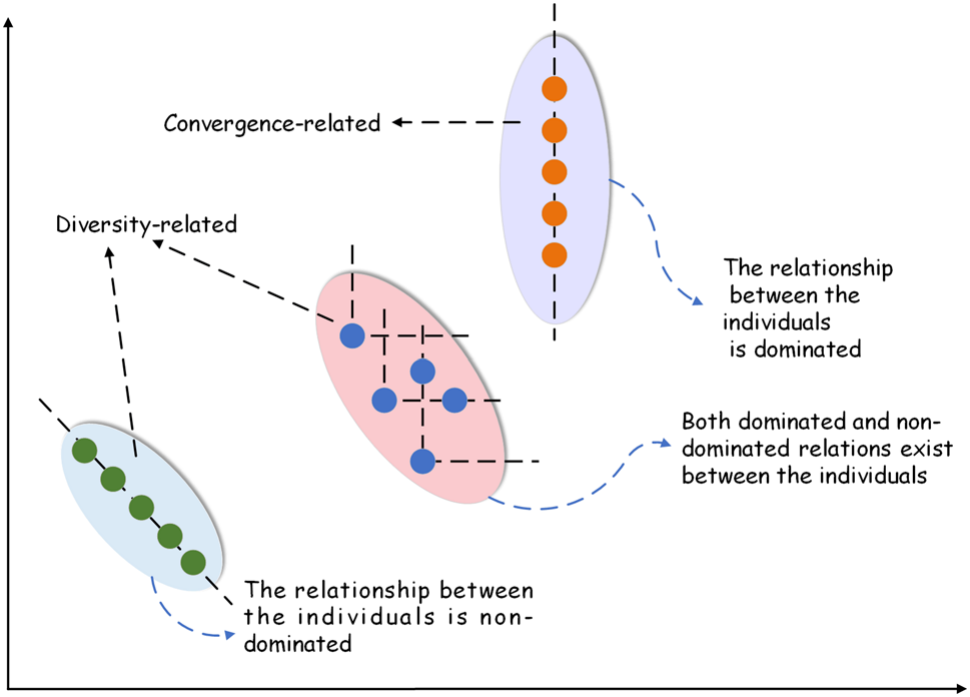
Classification of dominance relationships.

Among them, category a as well as category b are defined as diversity-related variables, while category c is defined as convergence-related. The classification after perturbation is shown in Fig. 2. Consequently, the key argument for solving DMOPs is to determine the type of decision variables.

**Figure 3:**
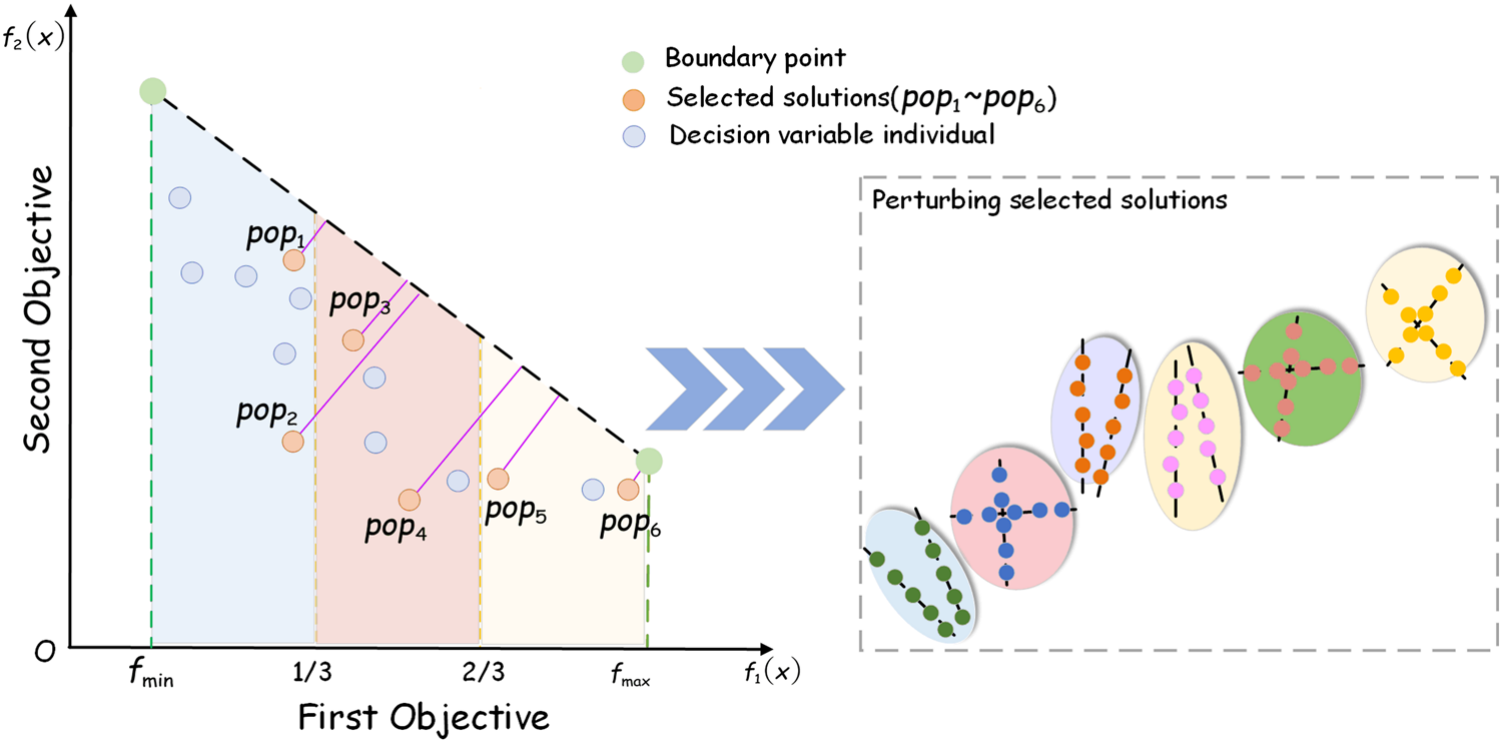
Points are selected from the decision space and perturbed.

This section presents a classification method for decision variables in static environments, as shown in Fig. 3:

Firstly, representative variables should be selected in the decision space. For the decision space in two-dimensional coordinates, the extreme value points on each objective value are found and defined as boundary points. Considering the first objective value, the region from the minimum of the boundary point to the maximum of the boundary point is evenly divided into three equal parts, in each region the nearest non-dominated point to the line of the boundary point and the farthest non-dominated point are selected respectively (if there is no point in the region then two points are randomly generated). In the decision space a total of 6 decision variables are selected and the selected decision variables are perturbed (the number of perturbations is 5).

**Figure 4:**
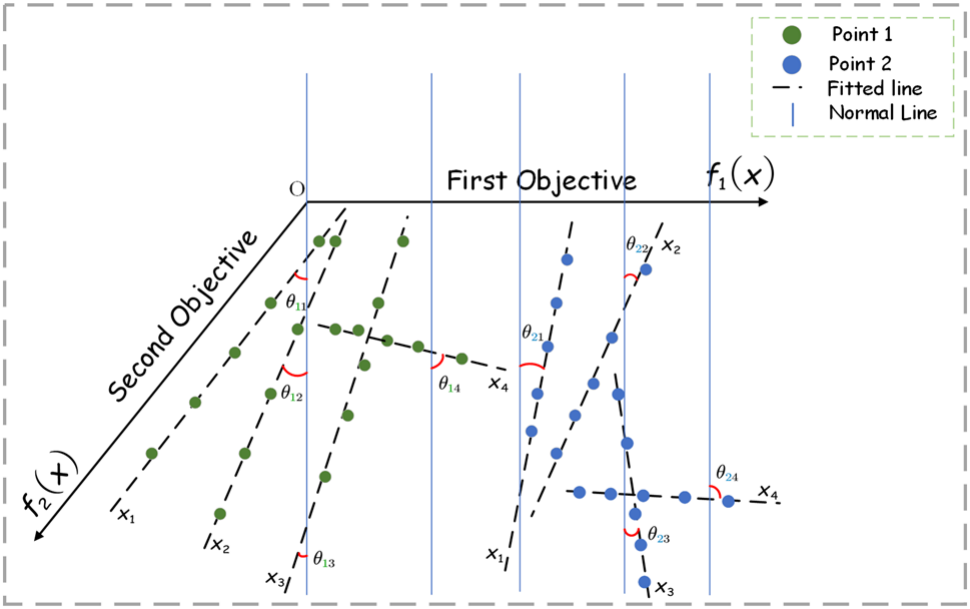
Convergence-related and diversity-related variables are identified using angles.

**Figure 5:**
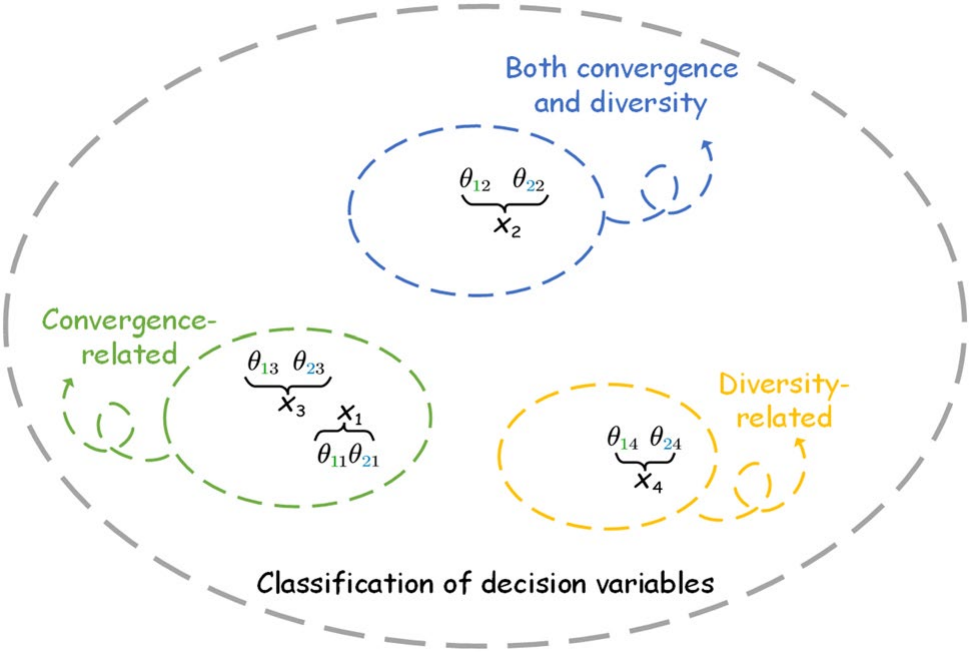
Three categories are classified according to the angle: diversity correlation, convergence correlation, and both diversity convergence correlation.

In order to be able to determine the class of the decision variables, the values of the perturbation points generated by the decision variables are normalized and the normalized points are fitted to a reference line $L_{fitted}$. We consider that the normal to the hyperplane can represent the direction of convergence, so the normal $L_{normal}$ to the hyperplane $f_1+f_2+\cdots f_m=1$ (*m* representing the number of objectives) is computed. As shown in Fig. 4, for the reason of clear presentation of the angle we make an example with two selected obtained points. Points 1 and 2 are represented by green dots and blue dots, respectively, in the figure. The decision variables $x_1, x_2, x_3, x_4$ are separately perturbed, and the fitted line $L_{fitted}$ and the normal line of hyperplane $L_{normal}$ will form 8 different angles after perturbation (only acute angles are chosen here). The angle is denoted as $\theta _{ij}$, where *i* represents the selected point and *j* indicates the pinch mark. Whether the decision variable $x_1$ is related to convergence can be determined by the angles. Theoretically, such judgments can be made by summing all $\theta _{i1}$ and taking the mean value, and the smaller $\overline{\sum _i{\theta _{i1}}}$ means that $L_{fitted}$ is closer to $L_{normal}$. Then the decision variable $x_1$ is determined to be related to convergence, otherwise it is related to diversity. At a brief level, the larger of the angle contributes more to diversity and the smaller of the angle contributes more to convergence. Since some decision variables have both diversity and convergence, in order to avoid overly absolute division, $k-means$ is used here to divide the correlation angles of decision variables $x_1, x_2, x_3$ and $x_4$ into three categories. As shown in Fig. 5, the four decision variables given are divided into three different categories. Algorithm 2 presents the detailed steps of decision variable category classification.

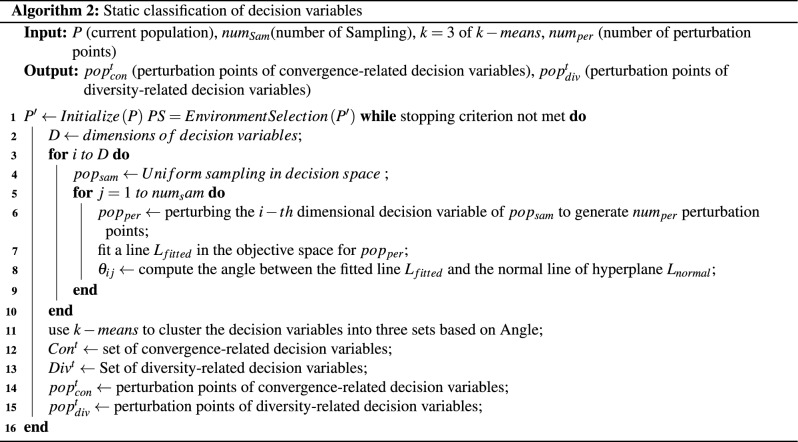



### Classification strategies in dynamic environments

Environmental change is an important element in DMOPs that has an impact on the evolution of populations, and it is the change in the environment that leads to problems such as goal conflict and consequently the problem of responding during the evolution of the population. In the absence of environmental changes, population convergence can only reach the optimal state in the current environment. However, in order to prevent the population from being trapped in an optimum state and failing to respond to environmental changes, the categorization of decision variables needs to be adjusted in dynamic environments. This adaptive adjustment of decision variable categorization enables populations to efficiently and effectively respond to changes in their environment, thereby promoting their survival and evolution. Therefore, incorporating dynamic adjustments to decision variable categorization is crucial for population convergence in a changing environment. In a static environment, decision variables are divided into three categories: those related to diversity, those related to convergence, and those related to both diversity and convergence. The classification of decision variables in a static environment serves as a guide for the classification of decision variables in a dynamic environment. The classification of decision variables needs to be adjusted due to changes in the environment, and different decision variables correspond to different prediction strategies. In the case where a decision variable remains constant across several consecutive changing environments, it is possible that the variable may not change in the next environmental shift. Such variables can be considered similar variables and do not require initialization in the prediction process. When a variable undergoes significant changes between two consecutive environmental shifts, it can be identified as a variable with a longer prediction process. Conversely, when a decision variable shows no significant change between two consecutive environmental shifts, it is classified as a variable with a shorter prediction process. In response to environmental changes, we employ non-parametric *t*-tests to adjust the categorization of decision variables to adapt to different predictive strategies. The formula for the non-parametric *t*-test is as follows:

6 $$\begin{aligned} t-test_i=\frac{|\overline{x_i}\left( t \right) -\overline{x_i}\left( t-1 \right) |}{\sqrt{\frac{\left( Var\left( x_i\left( t \right) \right) \right) ^2-\left( Var\left( x_i\left( t-1 \right) \right) \right) ^2}{N}}} \end{aligned}$$

where $\overline{x_i}\left( t \right)$ indicates the mean value of the decision variable of point *i* at time *t*. $Var\left( x_i\left( t \right) \right)$ expresses the variance of the decision variable of point *i* at time *t*. $\beta$ (a predetermined threshold) is set as a criterion for testing the attributes of the decision variables. $\beta$ is set as a criterion to test the properties of the decision variable, and if $t-test_i\leqslant \beta$, then the decision variable is considered to remain essentially constant over two successive environmental changes, thus defining that this decision variable is not a similar variable. The points on these variables as well as the perturbation points are added directly to the new population without initialization. If $t-test_i>\beta$, then the decision variable needs to be further subdivided into diversity-related and convergence-related, in other words, it needs to be further determined what prediction strategy should be assigned to this decision variable. Further determination of the decision variables needs to be adjusted based on historical information. First assume that $C_{}^{t}$ is the centroid of the non-dominated solution set POS, which can be obtained by the following equation:

7 $$\begin{aligned} C_{}^{t}=\frac{1}{|P_{Non-dom}^{t}|}\sum _{x^t\in P_{Non-dom}^{t}}{x_{}^{t}} \end{aligned}$$

where $C_{}^{t}$ denotes the centroid of the non-dominated solution set at moment *t*. $P_{Non-dom}^{t}$ indicates the size of the population of non-dominated individuals at moment *t*. $x_{}^{t}$ is specified as a non-dominated individual at moment *t*. Then, the results of the classification of decision variables are observed historically. Subsequently, *n* points $X_{}^{t}\left[ i \right] \left( i=1,2,3\ldots n \right)$ are generated by modifying the *i*-th decision variable in the $C_{}^{t}$ to the value of the selected points as well as the value of the perturbed point on the *i*-th decision variable at moment $t-1$. The generated points are compared with the $C_{}^{t}$ for domination, and if $X_{}^{t}\left[ i \right]$ can dominate the $C_{}^{t}$, then the category of decision variable *i* s adjusted to $\left( Con^t \right) ^{\prime }$, otherwise to $\left( Div^t \right) ^{\prime }$. The corresponding selected points and perturbed points on the decision variables in $\left( Con^t \right) ^{\prime }$ and $\left( Div^t \right) ^{\prime }$. The corresponding selected points and perturbed points on the decision variables in $\left( Con^t \right) ^{\prime }$ and $\left( Div^t \right) ^{\prime }$ are composed into two point sets $\left( pop_{Con}^{t} \right) ^{\prime }$ and $\left( pop_{Div}^{t} \right) ^{\prime }$, respectively. The two sets are adapted to different prediction strategies, which will be described in the nex section. The detailed steps of dynamic classification of decision variables are shown in Algorithm 3.
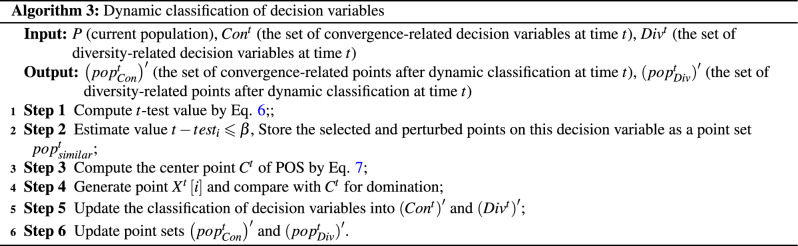


### Predictive strategy

As described in the section above, the selected points as well as the perturbed points are classified into three categories based on the classification of the decision variables:

1. Similar decision variables, directly add the point $pop_{similar}^{t}$ to the next iteration without initialization.
2. The set of points corresponding to the diversity-related decision variables $\left( pop_{Div}^{t} \right) ^{\prime }$ uses the following strategy:
  8 $$\begin{aligned} \begin{aligned}{}&dir=C_t-C_{t-1} \\&pop_{Div}^{t+1}=\left( pop_{Div}^{t} \right) \prime +rand*dir \end{aligned} \end{aligned}$$
  where *dir* indicates the step between the centroid at time *t*, and the centroid at time $t-1$, which is used to predict the $pop_{Div}^{t+1}$, $rand\in \left[ 0.5,1.5 \right]$ is a random value.
3. The prediction strategy for the point set $\left( pop_{Con}^{t} \right) ^{\prime }$ corresponding to the decision variables associated with convergence is as follows:
  9 $$\begin{aligned} pop_{Div}^{t+1}=\left( pop_{Con}^{t} \right) \prime +\frac{dir}{U_i\left( t \right) -L_i\left( t \right) }+Gaussian\left( 0,d \right) \end{aligned}$$
  where $U_i\left( t \right)$ and $L_i\left( t \right)$ represent the maximum and minimum values on the *i*th dimensional vector at time *t*, $Gaussian\left( 0,d \right)$ expresses a Gaussian perturbation with mathematical expectation dimension 0 and variance d.

## Test problems and performance indicators

### Test instances

We tested 12 benchmarks featuring the FDA1-FDA4^43^ test suite, the dMOP1-dMOP3^44^ test suite and the F5–F10^45^ test suite. Among them, FDA1-FDA4 are non-convex, continuous or discontinuous, time-varying as well as non-time-varying. FDA4 and F8 are three objectives test problems, the others are problems with two objectives. Both the FDA test suite and the DMOP test suite have linearly correlated decision variables, and non-linearly correlated decision variables are present in the F5–F10 test suite.

### Performance indicators

#### Inverted generational distance (IGD)

The metric under consideration represents a comprehensive evaluation metric, whereby its underlying calculation concept involves computing the minimum sum of distances that exist between individuals belonging to the actual POF and individuals generated by the algorithm under evaluation. It is noteworthy that the smaller the distance, the better the algorithm’s convergence and distribution performance. The IGD^46^ is calculated as follows:

10 $$\begin{aligned} \begin{aligned}{}&IGD\left( POF_t,P_t \right) =\frac{\sum \nolimits _{v\in POF_t}^{}{d\left( v,P_t \right) }}{|POF_t|}\\&d\left( v,P_t \right) =\min _{u\in P_t} \sqrt{\sum \nolimits _{j=1}^m{\left( f_{j}^{v}-f_{j}^{u} \right) ^2}} \end{aligned} \end{aligned}$$

At time *t*, $POF_t$ denotes the uniform distribution of the set of Pareto optimal points, and $P_t$ is the approximate set of $POF_t$. This can be interpreted as measuring the shortest distance between the true POF and the algorithm’s optimal solution. $d\left( v,P_t \right)$ represents the minimum Euclidian distance between *v* and the point in $P_t$.

#### The average of IGD (MIGD)

For DMOPs, MIGD provides a better evaluation of MOEAs. MIGD is expressed as:

11 $$\begin{aligned} MIGD=\frac{1}{|T|}\sum _{t\in T}{IGD\left( POF_t,P_t \right) } \end{aligned}$$

where *T* is a set of discrete time points in a run and |*T*| is the cardinality of *T*.

#### Hypervolume difference (HVD)

HVD^45^ is used to measure the distance between the hypervolume of POF obtained by the algorithm and the real POF. HVD can be calculated as:

12 $$\begin{aligned} HVD\left( POF_t,P_t \right) =HV\left( POF_t \right) -HV\left( P_t \right) \end{aligned}$$

where $HV\left( POF_t \right)$ represents the volume of the region in the target space enclosed by the set of non-dominated solutions and reference points obtained by the algorithm. the larger the HV value, the better the comprehensive performance of the algorithm. $P_t$ is an approximation set of POF at time *t*.

#### The mean of HVD (MHVD)

It can be set as:

13 $$\begin{aligned} MHVD=\frac{1}{|T|}\sum _{t\in T}{HVD\left( POF_t,P_t \right) } \end{aligned}$$

## Experimental results and analysis

### Parameter settings

In this paper, a comparison is made between the proposed algorithm DVC and six dynamic multi-objective algorithms, and all of them use the RM-MEDA algorithm in the optimization of the problem. The six compared algorithms are: feedforward prediction strategy (FPS), population prediction strategy (PPS), centroid and inflection point-based prediction strategy (CKPS), special point-based prediction strategy (SPPS), change response mechanism combining hybrid prediction and mutation strategy (HPPCM), and a prediction method based on diversity screening and special point prediction to solve dynamic optimization problems (DSSP). In order to compare the algorithms fairly, the parameters of each algorithm were basically set to the values of the parameters provided in the original paper, and some of the parameters were adjusted to ensure that they were compared in the same state as the present method. The experimental parameters are summarized as follows.

Each problem was tested 20 times independently, and 100 environmental changes were generated in each experiment. The degree of change of the environment $n_t$ is set to 10. The environment change frequency $\tau _t$ is set to 25. The number of iterations is 2500. The population size is 100 and the decision space dimension is 20.

- *Feed-forward prediction strategy (FPS)*^47^ The order in $AR\left( p \right)$ model was $p=3$, the number of cluster was set 5 and the maxi-mum length of history center point sequence was $M=23$. n the initial population, there are $3\left( m+1 \right)$ predicted points, 70% of the rest points are inherited from the previous population and the other 30% are randomly sampled from the search space.
- *Population prediction strategy (PPS)*^32^ PPS used the same $AR\left( p \right)$ model as FPS, and $p=3$, $M=23$.
- *A novel prediction strategy based on center points and knee points (CKPS)*^33^ The number of knee points is 9.
- *A predictive strategy based on special points (SPPS)*^48^ In the process of predicting the non-dominated set by feed-forward center points, set the Gaussian perturbation d to be 0.1.
- *Change response mechanism combining hybrid prediction and mutation strategy (HPPCM)*^49^ The autonomic evolution of the algebra $\varDelta t$ is set to 2.
- *A dynamic multi-objective evolutionary algorithm based on prediction (DSSP)*^42^ The number of knee points is 9. The number of perturbations is set to 5.

### Comparison of performance evaluation results

#### Analysis of FDA and dMOP evaluation results

Table 2 shows the average values along with the variance of MIGD for all compared algorithms on the FDA1-FDA4 and dMOP1-dMOP3 test problems. In the course of the experiment we ensured that the population underwent 100 environmental changes, 100 environmental changes were observed in three stages and assessed them using evaluation metrics. The first 20 environmental changes were identified as $1^{st}$ stage, the middle 40 environmental changes were recognized as $2^{nd}$ stage, and the last 40 were deemed to be $3^{rd}$ stage.

From an overall perspective, the DVC algorithm performs well in all phases, although it is slightly inferior to HPPCM in the FDA3 problem and the dMOP3 problem, but performs better in all other test problems, thus demonstrating that DVC has a strong ability to respond to environmental changes. FDA3 is a nonlinear test problem with large fluctuations in the set of Pareto solutions following environmental changes. Observing the characteristics of HPPCM, the HPPCM algorithm which uses the strategy of precisely controlling the polynomial mutation, this strategy effectively controls the updating and keeping of new populations. In contrast, our proposed DVC strategy relies more on the effect of decision variables in the population. This may be the reason why HPPCM outperforms DVC. dMOP3 test problem has no connection between the decision variables, which affects the results of DVC to a certain extent, but on closer inspection, it is easy to find that DVC is only slightly inferior to HPPCM, which also shows that DVC and HPPCM have their own advantages. HPPCM performed slightly better on the FDA3 issue, but DVC’s MIGD values were near parity with HPPCM. In the first stage, DVC was only marginally inferior to HPPCM for the FDA3 problem, and DVC was only a bit worse than the other methods for the dMOP problem, but performed well for FDA1, FDA2, FDA4, dMOP1, and dMOP2. This indicates that DVC is able to respond to environmental changes faster in the early stages of environmental changes. The MIGD values for the FDA2 problem in both phase 2 and phase 3 show superiority over the other five strategies.

Table 3 shows the MHVD metrics of seven different algorithms for the FDA and dMOP problems, and it can be seen from the table that the results of the seven different algorithms are similar to MIGD. Overall DVC has a strong competitive ability, especially in the FDA4 problem DVC’s results are obviously better than other algorithms.

DVC differs from CKPS, SPPS, HPPCM, and DSSP in that DVC does not focus on the selection and prediction of particular points, but more on the classification of decision variables and their changes in the environment. In CKPS, SPPS, HPPCM, and DSSP, these comparison algorithms adopt the strategy of special points, which are employed to increase the diversity of populations. Conversely, in contrast to randomly adding diversity to the population, increasing the diversity of the population using special points removes the instability of randomly adding special points. Nevertheless, the definition of special points is to some extent a coarse-grained definition. The advantage of DVC is that only the categories of decision variables need to be analyzed and then the attributes of decision variables are judged by historical information, and different attributes correspond to different prediction strategies, which can balance the diversity and convergence of populations.

**Table 2:** Mean and standard deviation of MIGD values of six strategies on FDA and dMOP test suites.

Problems	Statistic	FPS	PPS	CKPS	SPPS	HPPCM	DSSP	DVC
FDA1	Total	0.0525(0.01235)	0.0592(0.01680)	0.0297(0.00636)	0.0264(0.00630)	0.0211(0.00518)	0.0240(0.00843)	**0.0178(0.00413)**
	$1^{st}$stage	0.2137(0.06394)	0.2743(0.08625)	0.1168(0.03308)	0.1032(0.03278)	0.0813(0.02744)	0.0944(0.04377)	**0.0681(0.02205)**
	$2^{nd}$stage	0.0147(0.00159)	0.0100(0.00117)	0.0090(0.00013)	0.0082(0.00021)	0.0068(0.00076)	0.0072(0.00085)	**0.0066(0.00029)**
	$3^{rd}$stage	0.0136(0.00075)	**0.0063(0.00011)**	0.0089(0.00020)	0.0081(0.00022)	0.0067(0.00069)	0.0072(0.00092)	0.0070(0.00046)
FDA2	Total	0.0086(0.00069)	0.0093(0.00120)	0.0085(0.00056)	0.0087(0.00044)	0.0079(0.00087)	0.0080(0.00081)	**0.0074(0.00076)**
	$1^{st}$stage	0.0201(0.00298)	0.0219(0.00539)	0.0183(0.00276)	0.0170(0.00248)	0.0172(0.00419)	0.0167(0.00345)	**0.0160(0.00377)**
	$2^{nd}$stage	0.0060(0.00058)	0.0067(0.00058)	0.0065(0.00021)	0.0067(0.00025)	0.0058(0.00028)	0.0061(0.00098)	**0.0057(0.00008)**
	$3^{rd}$stage	0.0056(0.00011)	0.0059(0.00012)	0.0060(0.00010)	0.0068(0.00026)	0.0056(0.00004)	0.0058(0.00065)	**0.0052(0.00003)**
FDA3	Total	0.0638(0.00966)	0.0843(0.01313)	0.0383(0.00726)	0.0363(0.00724)	0.0275(0.00479)	0.0296(0.01232)	**0.0258(0.00647)**
	$1^{st}$stage	0.1971(0.04958)	0.2590(0.06128)	0.1222(0.03371)	0.1323(0.03870)	**0.0862(0.02561)**	0.1120(0.06379)	0.1023(0.03297)
	$2^{nd}$stage	0.0344(0.00824)	0.0436(0.01250)	0.0189(0.00279)	0.0137(0.00325)	**0.0064(0.00038)**	0.0140(0.00310)	0.0074(0.00060)
	$3^{rd}$stage	0.0298(0.00400)	0.0419(0.00778)	0.0179(0.00243)	0.0133(0.00262)	**0.0061(0.00035)**	0.0098 (0.00202)	0.0077(0.00067)
FDA4	Total	0.1421(0.00457)	0.1314(0.00333)	0.1184(0.00230)	0.1095(0.00211)	0.1020(0.00104)	0.1058 (0.00211)	**0.1011(0.00128)**
	$1^{st}$stage	0.1627(0.01336)	0.1609(0.00920)	0.1332(0.00845)	0.1300(0.00688)	0.1209(0.00692)	0.1208 (0.00504)	**0.1161(0.00498)**
	$2^{nd}$stage	0.1388(0.00416)	0.1258(0.00312)	0.1148(0.00268)	0.1050(0.00221)	**0.0969(0.00198)**	0.1019 (0.00224)	0.0976(0.00167)
	$3^{rd}$stage	0.1356(0.00490)	0.1230(0.00312)	0.1149(0.00232)	0.1043(0.00234)	0.0980(0.00245)	0.1026 (0.00414)	**0.0973(0.00216)**
dMOP1	Total	0.0080(0.00263)	0.1296(0.24085)	0.0076(0.00174)	0.0099(0.00276)	0.0068(0.00504)	0.0076 (0.00127)	**0.0064(0.00052)**
	$1^{st}$stage	0.0235(0.01367)	0.5522(1.06835)	0.0181(0.00901)	0.0310(0.01421)	0.0161(0.00382)	0.0188 (0.00900)	**0.0118(0.00272)**
	$2^{nd}$stage	**0.0043(0.00015)**	0.0528(0.09413)	0.0050(0.00005)	0.0050(0.00070)	0.0047(0.00004)	0.0050 (0.00004)	0.0051(0.00002)
	$3^{rd}$stage	**0.0043(0.00012)**	0.0057(0.00002)	0.0051(0.00006)	0.0048(0.00054)	0.0046(0.00006)	0.0050 (0.00003)	0.0051(0.00005)
dMOP2	Total	0.0617(0.01107)	0.0659(0.03602)	0.0267(0.00616)	0.0316(0.00584)	0.0252(0.00504)	0.0255 (0.00976)	**0.0223(0.00254)**
	$1^{st}$stage	0.2542(0.05699)	0.3008(0.16637)	0.1006(0.03204)	0.1263(0.03040)	0.1021(0.02651)	0.1035 (0.05015)	**0.0848(0.01365)**
	$2^{nd}$stage	0.0168(0.00106)	0.0142(0.01391)	0.0092(0.00012)	0.0093(0.00026)	**0.0069(0.00047)**	0.0071 (0.00070)	0.0075(0.00020)
	$3^{rd}$stage	0.0153(0.00080)	**0.0061(0.00003)**	0.0090(0.00020)	0.0090(0.00030)	0.0069(0.00042)	0.0068 (0.00034)	0.0073(0.00030)
dMOP3	Total	0.0528(0.01292)	0.0482(0.04869)	0.0277(0.00629)	0.0279(0.00828)	0.0242(0.00544)	**0.0240 (0.00901)**	0.0288(0.00431)
	$1^{st}$stage	0.2161(0.06577)	0.2173(0.00125)	0.1065(0.03292)	0.1111(0.04314)	0.0958(0.02797)	**0.0951 (0.04683)**	0.1204(0.02258)
	$2^{nd}$stage	0.0146(0.00143)	0.0099(0.00010)	0.0091(0.00017)	0.0082(0.00018)	0.0078(0.00138)	0.0073 (0.00063)	**0.0071(0.00047)**
	$3^{rd}$stage	0.0133(0.00065)	**0.0062(0.00954)**	0.0089(0.00011)	0.0081(0.00017)	0.0066(0.00043)	0.0071 (0.00082)	0.0069(0.00017)

The values which are in bold face donate to have the best effect of the six strategies.

**Table 3:** Mean and standard deviation of MHVD values of six strategies on FDA and dMOP test suites.

Problems	Statistic	FPS	PPS	CKPS	SPPS	HPPCM	DSSP	DVC
FDA1	Total	0.0965(0.01344)	0.0975(0.02015)	0.0465(0.00285)	0.0435(0.00435)	0.0374(0.00266)	0.0375(0.00462)	**0.0359(0.00481)**
	$1^{st}$stage	0.3668(0.06750)	0.4333(0.10000)	0.1562(0.01417)	0.1517(0.02257)	0.1321(0.01436)	0.1387(0.02547)	**0.1223(0.02471)**
	$2^{nd}$stage	0.0336(0.00371)	0.0218(0.00264)	0.0205(0.00038)	0.0179(0.00051)	0.0154(0.00117)	0.0158(0.00162)	**0.0148(0.00071)**
	$3^{rd}$stage	0.0310(0.00181)	**0.0135(0.00030)**	0.0203(0.00050)	0.0177(0.00050)	0.0146(0.00116)	0.0154(0.00211)	0.0180(0.00181)
FDA2	Total	0.0321(0.00084)	0.0320(0.00113)	0.0319(0.00074)	0.0322(0.00061)	0.0313(0.00068)	0.0313(0.00074)	**0.0311(0.00058)**
	$1^{st}$stage	0.0380(0.00434)	0.0380(0.00643)	0.0359(0.00386)	0.0361(0.00325)	0.0339(0.00354)	0.0329(0.00352)	**0.0323(0.00208)**
	$2^{nd}$stage	0.0306(0.00022)	**0.0302(0.00035)**	0.0308(0.00013)	0.0311(0.00014)	0.0306(0.00027)	0.0314(0.0004)	0.0319(0.00055)
	$3^{rd}$stage	0.0309(0.00007)	0.0310(0.00005)	0.0311(0.00007)	0.0314(0.00014)	0.0309(0.00003)	0.0312(0.00006)	**0.0305(0.00008)**
FDA3	Total	0.7923(0.02338)	0.8259(0.02868)	0.6657(0.00955)	0.6621(0.00654)	0.6583(0.00602)	0.6823(0.0098)	**0.6547(0.01009)**
	$1^{st}$stage	1.0789(0.08116)	1.1672(0.11775)	0.7527(0.02337)	**0.7379(0.02314)**	0.7657(0.02272)	0.7696(0.03068)	0.7720(0.03747)
	$2^{nd}$stage	0.7409(0.03579)	0.7667(0.03034)	0.6440(0.01583)	0.6420(0.01137)	**0.6308(0.00950)**	0.6683(0.01163)	0.6422(0.00801)
	$3^{rd}$stage	0.7076(0.01985)	0.7229(0.02694)	0.6461(0.01412)	0.6463(0.01333)	0.6347(0.00626)	0.6632(0.01132)	**0.6332(0.00324)**
FDA4	Total	0.4221(0.01725)	0.3799(0.01168)	0.3294(0.00692)	0.3024(0.00749)	0.2876(0.00799)	0.2894(0.00812)	**0.2739(0.00501)**
	$1^{st}$stage	0.5002(0.04865)	0.4877(0.03349)	0.3843(0.02738)	0.3749(0.02205)	0.3599(0.02345)	0.3521(0.02617)	**0.3311(0.02047)**
	$2^{nd}$stage	0.4096(0.01518)	0.3600(0.01229)	0.3158(0.00953)	0.2866(0.00816)	0.2654(0.00902)	0.2748(0.01034)	**0.2547(0.00541)**
	$3^{rd}$stage	0.3976(0.02063)	0.3486(0.00985)	0.3168(0.00773)	0.2838(0.00747)	0.2755(0.01146)	0.2769(0.01362)	**0.2589(0.01011)**
dMOP1	Total	0.1499(0.00093)	0.2199(0.12614)	0.1489(0.00106)	**0.1460(0.00146)**	0.1506(0.00059)	1.1472(0.00371)	0.1532(0.00039)
	$1^{st}$stage	0.1176(0.00467)	0.3376(0.38618)	0.1155(0.00565)	**0.0940(0.00766)**	0.1196(0.00300)	0.1161(0.00556)	0.1141(0.00150)
	$2^{nd}$stage	0.1576(0.00024)	0.2256(0.13406)	**0.1568(0.00025)**	0.1585(0.00074)	0.1579(0.00015)	0.1586(0.00035)	0.1581(0.00003)
	$3^{rd}$stage	0.1577(0.00030)	0.1582(0.00023)	**0.1568(0.00032)**	0.1581(0.00055)	0.1579(0.00012)	0.1582(0.00044)	0.1580(0.00013)
dMOP2	Total	0.2142(0.01691)	0.2253(0.04903)	0.1644(0.00351)	0.1677(0.00252)	0.1676(0.00185)	0.1657(0.00361)	**0.1639(0.00172)**
	$1^{st}$stage	0.4253(0.08359)	0.4708(0.21583)	0.1861(0.01909)	0.2018(0.01273)	0.1941(0.00985)	**0.1839(0.02431)**	0.1887(0.00625)
	$2^{nd}$stage	0.1664(0.00362)	0.1758(0.02352)	0.1596(0.00081)	0.1597(0.00085)	0.1616(0.00133)	0.1645(0.00189)	**0.1589(0.00137)**
	$3^{rd}$stage	0.1618(0.00260)	**0.1581(0.00024)**	0.1590(0.00043)	0.1596(0.00064)	0.1610(0.00124)	0.1633(0.00176)	0.1621(0.00168)
dMOP3	Total	0.0958(0.01522)	0.0869(0.01378)	0.0460(0.00447)	0.0431(0.00530)	**0.0343(0.00232)**	0.0357(0.00376)	0.0381(0.00263)
	$1^{st}$stage	0.3645(0.07608)	0.3797(0.06805)	0.1529(0.02389)	0.1499(0.02726)	**0.1172(0.01408)**	0.1200(0.00185)	0.1475(0.01231)
	$2^{nd}$stage	0.0336(0.00338)	0.0214(0.00272)	0.0208(0.00048)	0.0179(0.00043)	**0.0144(0.00107)**	0.0152(0.00180)	**0.0144(0.00071)**
	$3^{rd}$stage	0.0303(0.00150)	**0.0133(0.00027)**	0.0204(0.00030)	0.0175(0.00048)	0.0147(0.00088)	0.0149(0.00132)	0.0138(0.00053)

The values which are in bold face donate to have the best effect of the six strategies.

**Table 4:** Mean and standard deviation of MIGD values of six strategies on F5–F10.

Problems	Statistic	FPS	PPS	CKPS	SPPS	HPPCM	DSSP	DVC
F5	Total	0.3088(0.18713)	0.2816(0.11743)	0.0322(0.01004)	0.0319(0.00734)	0.0286(0.00739)	0.0346(0.01242)	**0.0269(0.00822)**
	$1^{st}$stage	1.0718(0.68643)	1.2651(0.54433)	0.0925(0.05217)	0.0866(0.03771)	0.0751(0.03516)	0.1081(0.06103)	**0.0653(0.04134)**
	$2^{nd}$stage	0.1916(0.17392)	0.0790(0.04525)	0.0181(0.00190)	0.0198(0.00196)	0.0164(0.00422)	**0.0154(0.00267)**	0.0232(0.00363)
	$3^{rd}$stage	0.0636(0.02009)	0.0170(0.00171)	0.0178(0.00130)	0.0182(0.00100)	0.0187(0.00654)	**0.0163(0.00342)**	0.0185(0.00193)
F6	Total	0.0527(0.02733)	0.0525(0.02698)	0.0210(0.00376)	0.0219(0.00705)	0.0189(0.00492)	0.0285(0.00555)	**0.0182(0.00277)**
	$1^{st}$stage	0.1247(0.12065)	0.1989(0.13472)	0.0468(0.01965)	0.0514(0.03669)	0.0572(0.02582)	0.0864(0.03281)	**0.0450(0.01413)**
	$2^{nd}$stage	0.0380(0.01241)	0.0216(0.00383)	0.0148(0.00037)	0.0148(0.00035)	**0.0098(0.00041)**	0.0144(0.00126)	0.0138(0.00081)
	$3^{rd}$stage	0.0331(0.00457)	0.0138(0.00061)	0.0149(0.00032)	0.0150(0.00041)	**0.0099(0.00054)**	0.0133(0.00061)	0.0144(0.00032)
F7	Total	0.1234(0.03443)	0.0923(0.04434)	0.0227(0.00803)	0.0212(0.00399)	**0.0166(0.00743)**	0.0243(0.01323)	0.0180(0.00266)
	$1^{st}$stage	0.3422(0.07395)	0.4108(0.22451)	0.0595(0.04207)	0.0504(0.02056)	0.0503(0.03906)	0.0974(0.07122)	**0.0417(0.01381)**
	$2^{nd}$stage	0.0835(0.04439)	0.0202(0.00377)	0.0139(0.00035)	0.0142(0.00031)	0.0088(0.00017)	**0.0086(0.00051)**	0.0104(0.00050)
	$3^{rd}$stage	0.0594(0.02857)	0.0130(0.00066)	0.0140(0.00036)	0.0143(0.00048)	**0.0085(0.00032)**	0.0090(0.00050)	0.0127(0.00064)
F8	Total	0.1440(0.00510)	0.1511(0.01251)	0.1560(0.00520)	0.1456(0.00738)	0.1289(0.00408)	**0.1141(0.00263)**	0.1335(0.00436)
	$1^{st}$stage	0.1969(0.02507)	0.2317(0.05906)	0.2117(0.02517)	0.2139(0.02998)	0.1791(0.02021)	0.1436(0.01444)	**0.1413(0.01599)**
	$2^{nd}$stage	0.1302(0.00413)	0.1330(0.00442)	0.1418(0.00546)	0.1292(0.00460)	0.1163(0.00311)	**0.1057(0.00209)**	0.1196(0.00352)
	$3^{rd}$stage	0.1325(0.00441)	0.1310(0.00411)	0.1437(0.00452)	0.1295(0.00525)	0.1178(0.00377)	0.1188(0.00162)	**0.1153(0.00063)**
F9	Total	0.4089(0.11057)	0.5884(0.20324)	0.1476(0.06633)	0.1019(0.02075)	0.0832(0.03398)	0.0723(0.01388)	**0.0644(0.01341)**
	$1^{st}$stage	1.1411(0.30435)	2.3269(1.01558)	0.4255(0.17298)	0.2950(0.08842)	0.2553(0.16235)	0.1266(0.03776)	**0.1110(0.02531)**
	$2^{nd}$stage	0.2311(0.09759)	0.2515(0.09232)	0.0818(0.06620)	0.0503(0.01248)	0.0456(0.02797)	0.0620(0.01999)	**0.0401(0.00680)**
	$3^{rd}$stage	0.2389(0.13232)	0.0993(0.06927)	0.0815(0.11799)	0.0618(0.02542)	0.0391(0.00733)	0.0451(0.01777)	**0.0386(0.00403)**
F10	Total	0.4312(0.05337)	0.5359(0.08500)	0.0860(0.02876)	**0.0729(0.01861)**	0.0744(0.01652)	0.1196(0.08332)	0.0931(0.00068)
	$1^{st}$stage	0.5899(0.10409)	1.6713(0.43441)	0.2988(0.14212)	0.2325(0.08635)	0.1824(0.08889)	0.1643(0.05256)	**0.1269(0.02338)**
	$2^{nd}$stage	0.4191(0.08585)	0.3076(0.10142)	0.0380(0.00789)	**0.0350(0.00558)**	0.0492(0.02139)	0.1479(0.18267)	0.0878(0.00785)
	$3^{rd}$stage	0.3679(0.07050)	0.2250(0.06185)	**0.0329(0.00298)**	0.0351(0.00771)	0.0483(0.00955)	0.0905(0.04511)	0.0944(0.01493)

The values which are in bold face donate to have the best effect of the six strategies.

#### Analysis of F5–F10 evaluation results

Tables 4 and 5 show the values of the MIGD and MHVD metrics on the F5–F10 test suite, respectively. It is clearly seen that HPPCM, DSSP, and DVC algorithms are much superior to the rest among the seven compared algorithms. Among these three algorithms, the better results of DVC prevailed. F5–F7 are nonlinearly correlated problems and DVC clearly exhibits better performance than HPPCM and DSSP comparison algorithms. The nonlinear correlation problem also implies that PF changes are complex and requires a more demanding strategy for responding to environmental changes. For the three-objective problem F8, DSSP outperforms DVC to some extent because DSSP takes more objectives into account when sampling at special points. but the results of DVC are not comparable to DSSP, and the values of MIGD outperform DSSP in the first and third stages. The resilience of the DVC approach is also demonstrated by the standard deviation values presented in Tables 2, 3, 4 and 5. These results reveal that the DVC method exhibits a remarkable robustness and can swiftly and efficiently adapt to environmental variations, displaying an exceptional ability to tackle problems of varying complexities with ease.

**Table 5:** Mean and standard deviation of MHVD values of six strategies on F5–F10.

Problems	Statistic	FPS	PPS	CKPS	SPPS	HPPCM	DSSP	DVC
F5	Total	0.5501(0.12626)	0.5124(0.07330)	0.2769(0.01312)	0.2782(0.01280)	0.2889(0.02210)	0.2750(0.01432)	**0.2725(0.01327)**
	$1^{st}$stage	1.2207(0.43228)	1.4519(0.28264)	0.4064(0.07001)	0.4155(0.06438)	0.4468(0.09702)	**0.3923(0.07002)**	0.4132(0.05877)
	$2^{nd}$stage	0.4677(0.14720)	0.3309(0.05948)	0.2466(0.00230)	0.2455(0.00379)	0.2516(0.01051)	0.2458(0.00332)	**0.2379(0.00468)**
	$3^{rd}$stage	0.3139(0.03985)	0.2477(0.00173)	0.2456(0.00171)	0.2457(0.00174)	0.2512(0.00779)	0.2444(0.00093)	**0.2403(0.00508)**
F6	Total	0.2997(0.04996)	0.3222(0.03755)	0.2659(0.00655)	0.2693(0.01432)	0.2725(0.01061)	0.2792(0.01264)	**0.2655(0.00412)**
	$1^{st}$stage	0.4839(0.20772)	0.6239(0.18580)	0.3462(0.03272)	0.3647(0.07603)	0.3867(0.05547)	0.4200(0.06458)	**0.3455(0.02249)**
	$2^{nd}$stage	0.2608(0.02877)	0.2545(0.00503)	0.2467(0.00117)	0.2467(0.00093)	**0.2453(0.00079)**	0.2460(0.00111)	0.2459(0.00118)
	$3^{rd}$stage	0.2510(0.00852)	0.2466(0.00144)	0.2470(0.00104)	0.2467(0.00095)	**0.2455(0.00050)**	0.2456(0.00090)	0.2472(0.00165)
F7	Total	0.4390(0.06145)	0.3596(0.03072)	0.2690(0.01654)	0.2664(0.00844)	0.2672(0.01441)	0.2718(0.01178)	**0.2614(0.00741)**
	$1^{st}$stage	0.8908(0.11463)	0.8307(0.15396)	0.3740(0.08647)	0.3560(0.04440)	0.3633(0.07469)	0.3857(0.06174)	**0.3332(0.03972)**
	$2^{nd}$stage	0.3541(0.08694)	0.2492(0.00359)	**0.2443(0.00101)**	0.2454(0.00071)	0.2444(0.00074)	0.2450(0.00120)	**0.2443(0.00105)**
	$3^{rd}$stage	0.3093(0.05246)	0.2463(0.00111)	**0.2439(0.00080)**	0.2447(0.00093)	0.2443(0.00075)	0.2444(0.00068)	0.2444(0.00094)
F8	Total	0.3810(0.01471)	0.3954(0.03307)	0.4108(0.02681)	0.3852(0.01921)	0.3223(0.01427)	**0.2946(0.00416)**	0.3277(0.00396)
	$1^{st}$stage	0.5525(0.06016)	0.6076(0.16045)	0.5809(0.13203)	0.5864(0.08519)	0.4663(0.05926)	**0.3907(0.02803)**	0.4568(0.02104)
	$2^{nd}$stage	0.3375(0.01248)	0.3504(0.01283)	0.3684(0.05066)	0.3374(0.00970)	**0.2889(0.00725)**	0.2966(0.00718)	0.2959(0.00706)
	$3^{rd}$stage	0.3431(0.01531)	0.3397(0.01085)	0.3725(0.02345)	0.3373(0.01247)	0.2872(0.01162)	**0.2736(0.00841)**	0.2983(0.00646)
F9	Total	0.6661(0.11342)	0.6408(0.07758)	0.3705(0.03890)	0.3411(0.01947)	0.3353(0.04994)	0.3165(0.01833)	**0.3113(0.02232)**
	$1^{st}$stage	1.2766(0.20275)	1.6206(0.23861)	0.7310(0.18668)	0.6319(0.06566)	0.6164(0.12440)	**0.4923(0.06364)**	0.5565(0.10975)
	$2^{nd}$stage	0.5151(0.11523)	0.4945(0.09275)	0.2950(0.01834)	0.2697(0.01675)	0.2695(0.05153)	0.2849(0.02556)	**0.2593(0.00953)**
	$3^{rd}$stage	0.5271(0.18101)	0.3216(0.07873)	0.2746(0.00704)	0.2744(0.02416)	0.2674(0.02417)	0.2647(0.01501)	**0.2468(0.01399)**
F10	Total	0.8915(0.07819)	0.7720(0.06904)	0.3612(0.01334)	**0.3361(0.02204)**	0.4034(0.05090)	0.3745(0.05411)	0.3927(0.03353)
	$1^{st}$stage	1.2343(0.13459)	1.5730(0.10988)	0.7354(0.05476)	0.6343(0.10452)	0.6220(0.11651)	**0.4889(0.09201)**	0.5665(0.09724)
	$2^{nd}$stage	0.8426(0.12312)	0.6341(0.13136)	0.2782(0.01809)	**0.2652(0.00560)**	0.3423(0.04547)	0.3598(0.09463)	0.3631(0.01942)
	$3^{rd}$stage	0.7776(0.09777)	0.5294(0.08679)	0.2666(0.01608)	**0.2653(0.00905)**	0.3606(0.06961)	0.3350(0.0596)	0.3398(0.03363)

The values which are in bold face donate to have the best effect of the six strategies.

### Distribution diagram of final population

**Figure 6:**
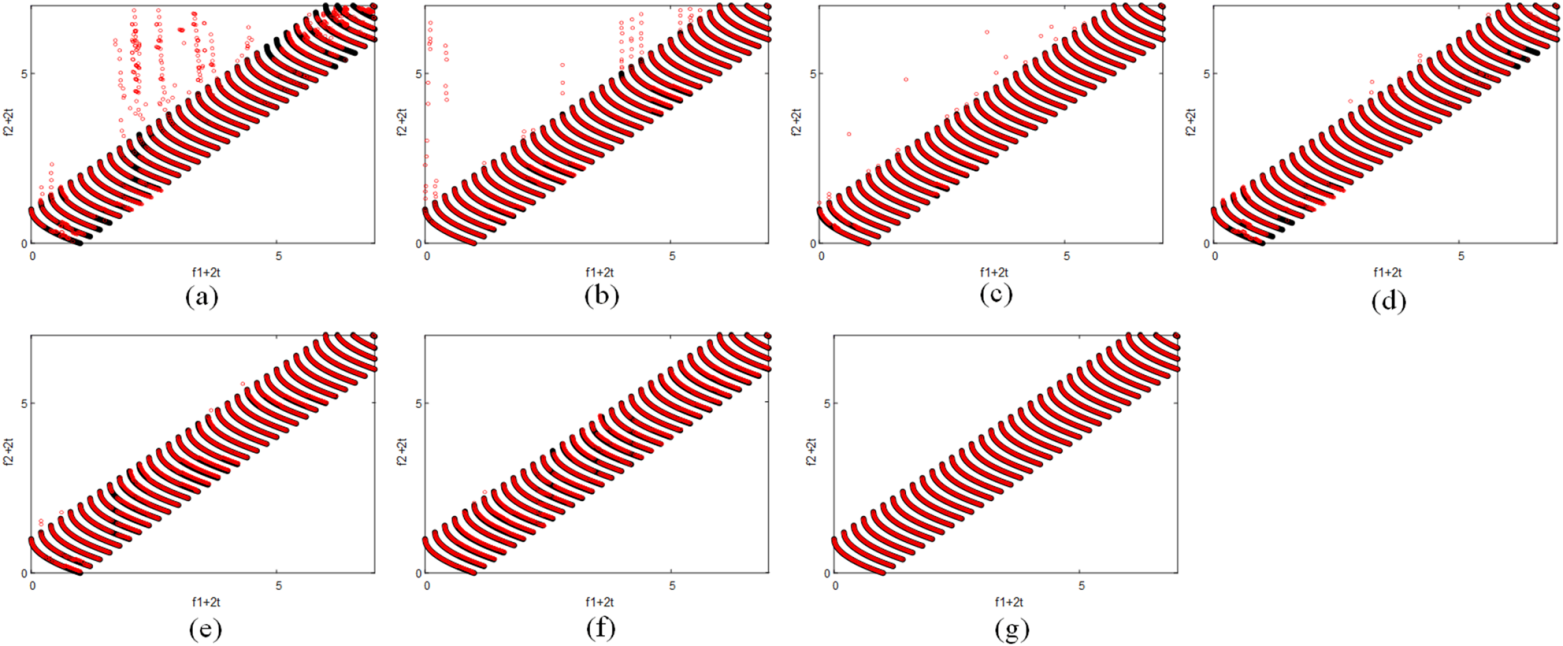
(**a**–**g**) exhibit the results of algorithms FPS, PPS, CKPS, SPPS, HPPCM, DSSP, and DVC with $n_t=10$ and $\tau _t=25$ on the FDA1 problem, respectively.

**Figure 7:**
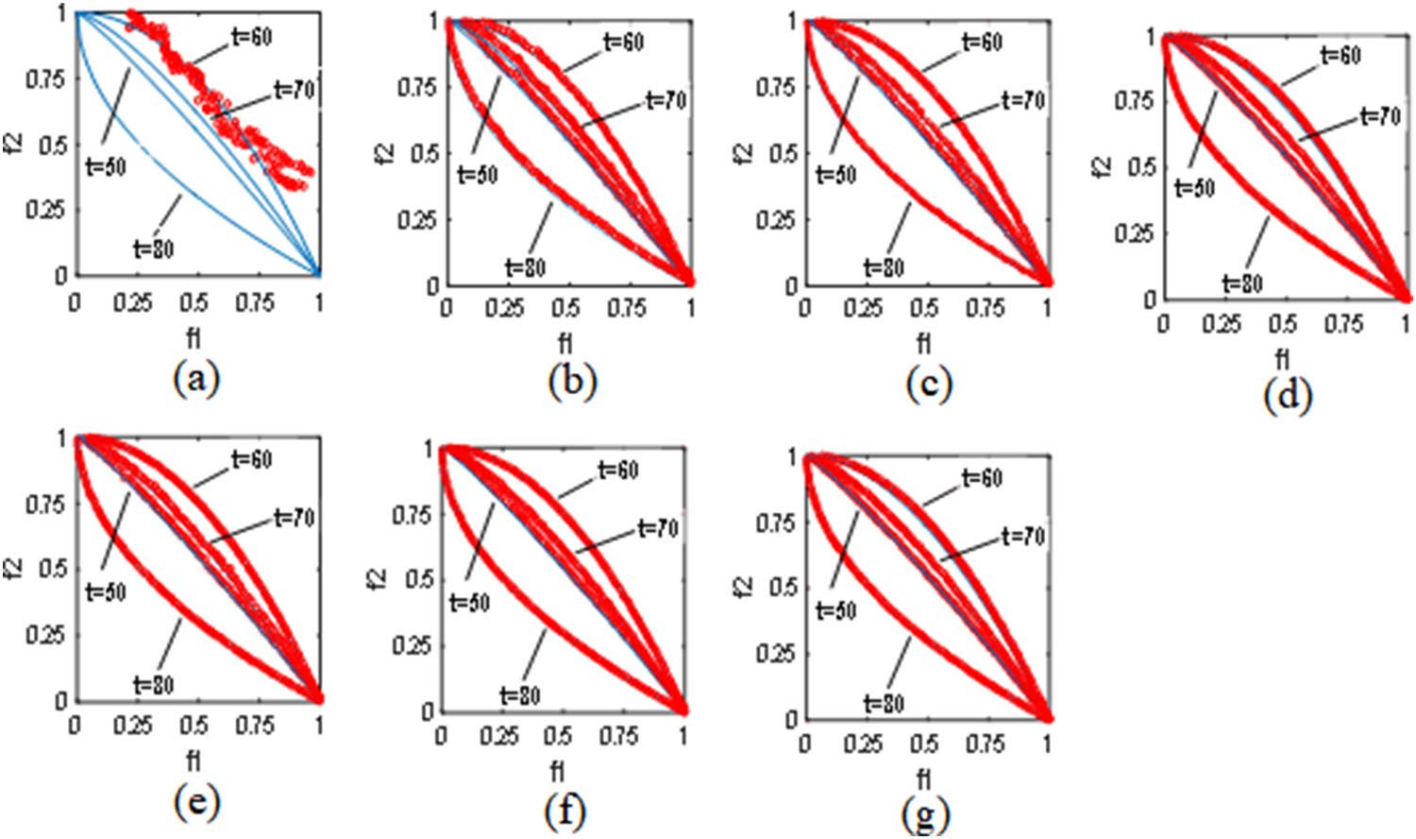
(**a**–**g**) exhibit the results of algorithms FPS, PPS, CKPS, SPPS, HPPCM, DSSP, and DVC with $n_t=10$ and $\tau _t=25$ on the dMOP2 problem at the late stage of environmental changes, respectively.

Figure 6 visualizes the tracking ability of the POF on the FDA1 problem for DVC and the other six comparison algorithms. It can be seen from Fig. 6 that DVC has a more uniform POF distribution compared to the other six comparison algorithms, which also shows that the DVC algorithm is able to better balance diversity and convergence. The dMOP2 problem has the qualities of a changing POF as well as a changing POS. On the dMOP2 problem, we have selected different algorithms to demonstrate the late state of the changing environment, and it can be seen in Fig. 7 that most of the algorithms basically track the POF well, but DVC demonstrates a more superior performance on the uniform distribution.

**Table 6:** Mean and standard deviation of MIGD values and MHVD values of HPPCM and DVC on different $\tau _t$ values.

Problems	$\left( \tau _t,n_t \right)$	MIGD		MHVD	
		HPPCM	DVC	HPPCM	DVC
FDA1	(20,10)	0.0370(0.00691)	**0.0340(0.00235)**	0.0528(0.00326)	**0.0469(0.00313)**
	(25,10)	0.0211(0.00518)	**0.0178(0.00413)**	0.0374(0.00266)	**0.0359(0.00481)**
	(30,10)	0.0161(0.00531)	**0.0154(0.00317)**	**0.0283(0.00265)**	0.0303(0.00377)
FDA2	(20,10)	0.0087(0.00908)	**0.0082(0.00034)**	0.0326(0.00105)	**0.0315(0.00044)**
	(25,10)	0.0079(0.00087)	**0.0074(0.00076)**	0.0313(0.00068)	**0.0311(0.00058)**
	(30,10)	**0.0072(0.00074)**	0.0073(0.00296)	**0.0307(0.00040)**	0.0309(0.00036)
FDA4	(20,10)	0.1094(0.00244)	**0.1082(0.00119)**	0.2986(0.01041)	**0.2934(0.00405)**
	(25,10)	0.1020(0.00104)	**0.1011(0.00128)**	0.2876(0.00799)	**0.2739(0.00501)**
	(30,10)	**0.0962(0.00129)**	0.0964(0.00165)	**0.2473(0.00487)**	0.2544(0.00545)
dMOP1	(20,10)	**0.0082(0.00175)**	0.0084(0.00118)	0.1510(0.00152)	**0.1503(0.00066)**
	(25,10)	0.0068(0.00504)	**0.0064(0.00052)**	**0.1506(0.00059)**	0.1532(0.00039)
	(30,10)	0.0062(0.00120)	**0.0061(0.00044)**	0.1508(0.00054)	**0.1504(0.00044)**
dMOP2	(20,10)	0.0384(0.00724)	**0.0374(0.00904)**	0.1754(0.00443)	**0.1644(0.00216)**
	(25,10)	0.0252(0.00504)	**0.0223(0.00254)**	0.1676(0.00185)	**0.1639(0.00172)**
	(30,10)	0.0196(0.00645)	**0.0168(0.00458)**	0.1656(0.00429)	**0.1593(0.00404)**
F6	(20,10)	0.0252(0.00519)	**0.0248(0.00261)**	0.2811(0.01186)	**0.2729(0.00377)**
	(25,10)	0.0189(0.00492)	**0.0182(0.00277)**	0.2725(0.01061)	**0.2655(0.00412)**
	(30,10)	0.0144(0.00207)	**0.0127(0.00106)**	0.2611(0.00575)	**0.2603(0.00321)**
F9	(20,10)	0.1030(0.01668)	**0.0864(0.01512)**	0.3551(0.01192)	**0.3419(0.02853)**
	(25,10)	0.0832(0.03398)	**0.0644(0.01341)**	0.3353(0.04994)	**0.3113(0.02232)**
	(30,10)	**0.0658(0.01456)**	0.0731(0.02148)	0.3079(0.01038)	**0.3014(0.01270)**

Significant values are in bold.

## Discussion

In order to examine the effectiveness and robustness of the algorithm, we conducted a specific comparison with its formidable competitor, HPPCM. In our experiment, we maintain the value of $n_t$ unchanged while observing the algorithm’s adaptability to dynamic environments by varying the value of $\tau _t$, which represents the frequency of environmental changes. From the table 6, it can be clearly seen that DVC outperforms HPPCM significantly in both MIGD and MHVD metrics. This further illustrates that DVC is more capable of adapting to environments with varying frequencies of changes. During 100 phased environmental changes, HPPCM indeed demonstrates comparable results to DVC. However, when altering the frequency of environmental changes, DVC exhibits a certain advantage. HPPCM’s precise and controllable abrupt mutation strategy indeed holds some advantage under highly changing environments, whereas DVC’s stability relies on the relationships between decision variables, demonstrating superiority under different environmental change frequencies. Both algorithms have their respective strengths and weaknesses, but DVC’s stability stands as a robust competitive advantage.

## Conclusions and future work

The role of decision variables in environmental change is fully considered in this paper, and a DMOP-DVC based on decision vector classification is proposed by integrating the historical information of static classification of decision variables. The proposed method reflects the evolutionary direction for decision variables in the current environment, and different classes of prediction strategies are used to optimize the evolution of populations to balance convergence and diversity.

From the experiments, many advantages are shown on DMOP-DVC, but the algorithm also has some drawbacks. For example, the accuracy of prediction can be further improved in the prediction strategy, and future work can consider introducing some training models to consider global historical changes. If the environment changes little or the environment changes drastically, whether different prediction strategies are needed to cope with it, these are the future works that are worth looking forward to.

## Data Availability

The datasets used during the study are available from the corresponding author upon reasonable request.
